# Exploring diverse applications of team-based care in preventive medicine: a scoping review

**DOI:** 10.1186/s12875-026-03311-8

**Published:** 2026-04-29

**Authors:** Van Nguyen, Swetha S Kumar, Sophia Reyes, Jacky Zhao, Chirk Jenn Ng

**Affiliations:** 1 SingHealth Family Medicine Residency Programme, Singapore, Singapore; 2 SingHealth Centre for Population Health Research and Implementation (CPHRI), Singapore, 150167 Singapore; 3 Ministry of Health Holdings, Singapore, Singapore; 4 Duke-NUS Medical School, Singapore, 150167 Singapore; 5 SingHealth Polyclinics, Singapore, Singapore

**Keywords:** Preventive medicine, Team-based care, Scoping review, Systematic review, Integrated care, Primary care

## Abstract

**Background:**

Preventive medicine is essential for improving population health and reducing health care costs. Team-based care (TBC) is increasingly recognised as a promising approach to enhance outcomes for chronic diseases and improving care experiences. However, a comprehensive review of TBC in preventive medicine is lacking.

**Aim:**

This scoping review aimed to provide an overview of currently available TBC models in preventive care, including their composition, structure and delivery methods. Additionally, it sought to explore the qualitative outcomes such as perceptions of patients and health care providers, along with TBC-related processes.

**Methods:**

Following the Arksey and O’Malley Framework, relevant articles were searched and selected from databases including PubMed, MEDLINE, Embase, CINAHL, Scopus and PsycINFO, covering records published from inception to March 2023. Relevant data from the selected articles were then extracted and synthesised. Studies implementing TBC approaches for preventive health services were included.

**Results:**

A total of 6779 titles and abstracts were screened; 92 studies were included in the final analysis. Preventive health services focused on disease screening, vaccination, maternal and child health, and chronic disease care. The TBC models used varied (including multidisciplinary teams and patient-centred homes). The common outcomes were TBC delivery, implementation, health care providers’ perceptions of TBC practices, their roles as well as patients’ satisfaction.

**Conclusion:**

While TBC is widely implemented in preventive medicine, significant variability exists in team organisation across conditions and settings. Future research should focus on systematically evaluating the applicability and efficacy of currently available TBC models in different preventive care settings.

**Supplementary Information:**

The online version contains supplementary material available at 10.1186/s12875-026-03311-8.

## Key terms and definitions

General Preventive Services: Preventive care services not described in sufficient detail for subcategory classification, or involving multiple service types.

Pediatric Care: Preventive services targeting children and adolescents, including well-child visits, developmental screenings, and age-appropriate immunizations.

Geriatric Care: Preventive services targeting older adults (≥ 65 years), including functional assessments, fall prevention, and age-appropriate screenings.

Maternal, Adolescent and Child Health: Preventive services targeting maternal, adolescent, and child populations, including antenatal care, postnatal support, and health interventions aimed at promoting the well-being of mothers, adolescents, and young children.

Mental Health: Preventive services focused on mental and behavioral health, including screenings, early intervention, and counseling across the lifespan.

Screening and Health Promotion/Weight Management: Clinical screenings (e.g., cardiovascular, cancers, mental health), obesity and weight management programs, health education, and vaccination initiatives.

Prevention/Management of High-Risk Lifestyle Activities: Interventions targeting specific high-risk behaviors, including tobacco use, alcohol misuse, unsafe sexual practices, physical inactivity, and unhealthy dietary patterns.

Clinical staff: Health professionals directly involved in patient care, including but not limited to, primary health care providers, nurses, specialists, allied health care professionals (e.g., health educators/counsellors, health promotion officers, podiatrists, optometrists, physical therapists, occupational therapists, neonatologists).

Community partners: Non-clinical collaborators supporting care delivery, including case workers, managers, social workers, liaisons, volunteers, physical activity educators, referral coordinators, parent advisors, caregivers.

Support staff: Personnel who provide logistical, administrative, or technical support within care settings, such as medical assistants, data coordinators, front desk staff, office managers, lab assistants, and administrative support.

Task separation: A care model where roles and responsibilities are clearly delineated with minimal overlap.

Task sharing: A collaborative model where multiple team members jointly perform or alternate responsibilities.

Task shifting: The delegation of clinical responsibilities from higher-trained professionals to those with less formal training, under appropriate supervision.

## Introduction

### Background

Team-based care (TBC), defined as care provided by “two or more health care professionals who work collaboratively with patients and their caregivers to accomplish shared goals” [1], is increasingly recognised as a promising care delivery approach to improving both health outcomes and patient and care provider experiences [2, 3] Specifically, it has been shown to improve chronic care for hypertension and depression [4–6]. Regarding care experiences, patients feel understood and supported by a care team [2], while healthcare providers could improve their overall well-being by leveraging the roles of each team member, building mutual respect and relationship [7, 8].

Besides from its implementation in chronic care delivery, TBC’s role has increasingly been explored in the context of preventive care services [9–11] This push is partly attributed to the increasing emphasis on health prevention and promotion, demand for task shifting to relieve the heavy clinician’s workload, and rising healthcare cost [12]. However, there is a wide variation in how it is implemented and inconsistent outcomes regarding its effectiveness in improving preventive service uptake and enhancing experiences of patients and care providers. For example, while a USA study conducted in family practice found the involvement of non-physician staff in patient education improved colorectal cancer screening rates [13], another TBC intervention in Quebec, Canada employing teams of physicians, nurses and other health professionals did not show any detectable differences in screening rates [14]. Factors that might explain these variations include team composition, communication strategies, collaboration structures, and available health care resources [1, 15–17].

Therefore, this scoping review aimed to provide an overview of existing TBC models in preventive care, including their composition, structure and delivery methods, as well as to characterize the outcomes such as uptake of preventive services, experiences of stakeholders and TBC-related processes relevant to the team-based intervention.

## Methods

This scoping review was guided by the Arksey and O’Malley Framework and the methodology described by Levac et al [18, 19]. The Preferred Reporting Items for Systematic Reviews and Meta-Analyses–Extension for Scoping Reviews (PRISMA-ScR) guideline was followed. (Supplemental File 1) No protocol was registered.

### Eligibility criteria

All studies implementing preventive health services using any team-based, integrated approaches were included. TBC was defined earlier in this review. Preventive care includes health promotion and disease prevention, understood as “specific, population-based and individual-based interventions for primary and secondary prevention, aiming to minimise the burden of diseases and associated risk factors” [20]. In terms of study type, the review included only primary studies; it excluded protocols, reviews, meta-analyses and commentaries. This review also excluded studies: using vertical integration of care whereby hospital-based specialists collaborated with primary care providers (PCPs); involving collaboration with other non-medical fields such as dentistry; focusing on the management of chronic diseases and/or their complications; aiming to prevent hospital admissions/readmissions or optimise risk factors in patients already diagnosed with a condition; evaluating any medication-related intervention except vaccinations; and focusing on exploring the experiences of a single member of the care team and/or perceptions of other members in their specific role, rather than as a team member.

### Information sources and search strategy

The systematic literature search was conducted in PubMed, Embase, CINAHL, Scopus and PsycINFO. We searched these databases from their inception to 13 Mar 2023 (and subsequently updated until 10 Jan 2026) for relevant English-language articles. We developed the search strategy based on three concepts: preventive health services, integrated care and primary care. A medical librarian guided the development of the search strategy and the Covidence software was used to manage the scoping review [21].

The full search strategy can be found in Appendix S1. Both subjective headings and keywords were used. We did not search the grey literature, conduct a hand search, or contact authors for additional information regarding their studies.

### Study selection

Two independent reviewers (VN and SR) screened the titles and abstracts of all citations based on the inclusion and exclusion criteria. Full texts were then retrieved and screened independently by both reviewers. An independent third reviewer (CJN) was consulted when both researchers were unable to come to an agreement at both stages. Any disagreement was discussed until a consensus was reached.

### Data charting

Article screening and data extraction were conducted using a standardised protocol. A data extraction template, adapted from the Cochrane Data Collection Form for Intervention Reviews for RCTs and Non-RCTs (Available as Supplement File 2), was used to extract the necessary information from the retrieved full texts. Two reviewers (VN and JZ) extracted and entered the data into the data extraction template, and a third reviewer (SSK) verified the accuracy of the extracted data.

### Data items

Since we were interested in both the TBC processes and outcomes, the outcomes were categorised as process (experiences of relevant stakeholders), clinical/health (disease-related indicators) and TBC (team size, composition and description) outcomes. Other data recorded were: authors, publication year, objectives, targeted health condition, study design, setting, population, data collection methods and preventive health service.

### Result synthesis

The results of this review were classified into three stages based on the methodology described by Levac et al.: (a) analysis, which included descriptive and numerical analyses; (b) reporting, which involved presenting the results relevant to the research question; and (c) interpretation, which involved considering the meaning of the findings in relation to the overall study purpose and their implications for future research, practice and policy [19].

### Consultation exercise

A regular consultation exercise was conducted with an expert in the field of primary care (CJN). Preliminary findings were presented and discussed at a symposium during a scientific congress, where feedback was received from attending primary care physicians and researchers.

## Results

### Screening results

**Fig. 1:**
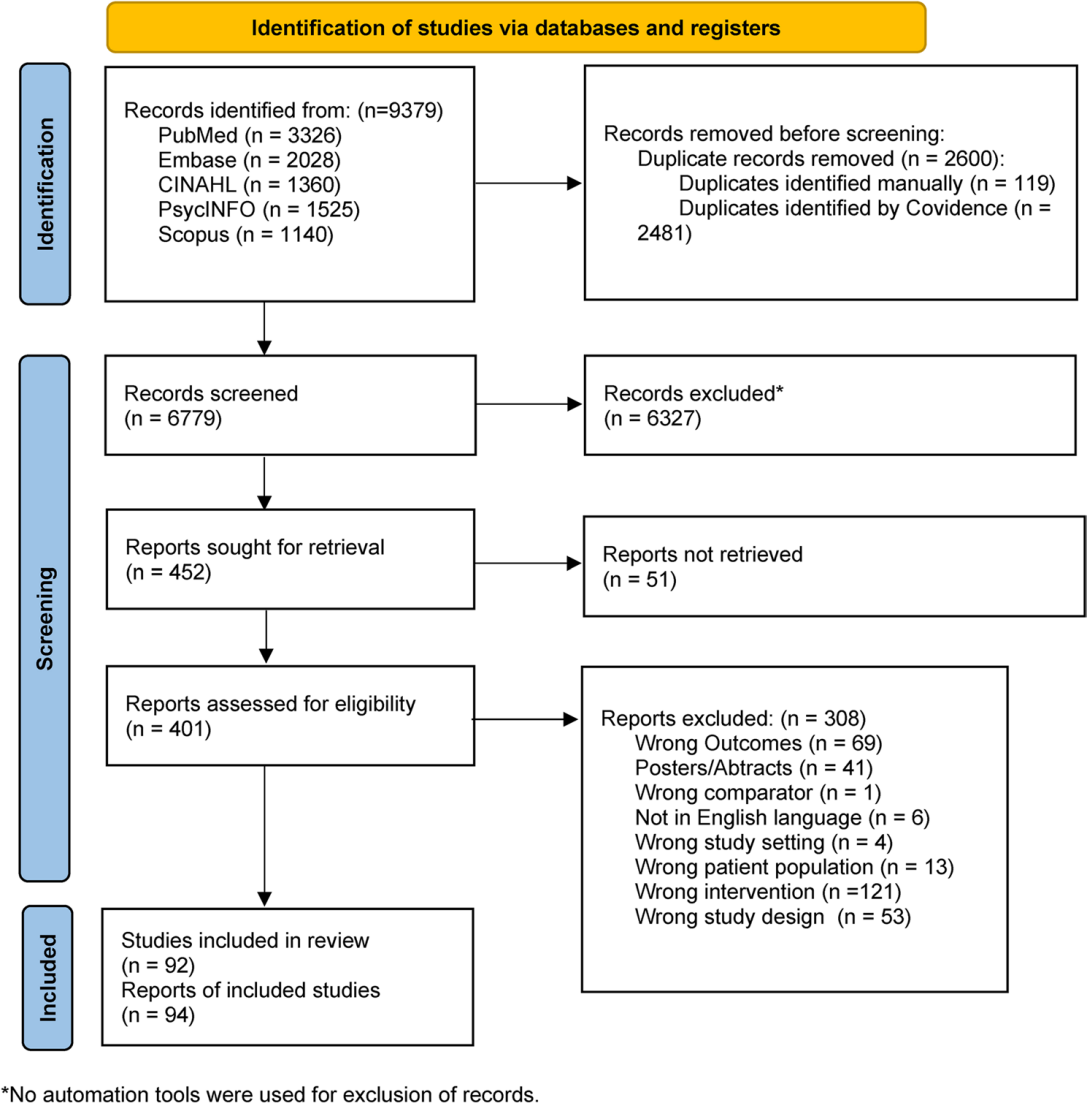
Preferred reporting items for systematic reviews and meta-analyses–extension for scoping reviews flowchart. *No automation tools were used for exclusion of records

Of 6779 titles and abstracts screened, 452 full texts were identified for screening; however, 51 were irretrievable. Among the retrieved articles, 308 were excluded. A total of 94 articles were included in the final analysis (1 additional article retrieved from an included analysis paper of the same intervention). The screening process and results are detailed in the PRISMA-ScR flowchart illustrated in Fig. 1.

Key characteristics of included studies were presented in Table 1.

**Table 1:** Characteristics of included studies

Study	Team Composition	Main Leaders	TBC Model	Types of Communication	Task Assignment
Abbasi et al. 2021 [9]	Clinical staff + Community partners + Caregiver	PCP	Multidisciplinary team (Member(s) led)	Unclear	Separation
Aboueid et al. 2018 [22]	Clinical staff + Community partners	Unclear	Multidisciplinary team	Unclear	Separation
Aboueid et al. 2018 [23]	Clinical staff + Community partners	Unclear	Multidisciplinary team	Unclear	Separation
Abrams et al. 2015 [24]	Clinical staff + Community partners	Unclear	Multidisciplinary team	Information exchange	Sharing and Shifting
Ammerman et al. 2022 [25]	Clinical staff + Community partner + Caregiver	Unclear	Multidisciplinary team	Unclear	Separation
Arthur NSM et al. 2023 [26]	Clinical staff + Support staff	Unclear	Multidisciplinary team (Medical home)	Unclear	Unclear
Ashcroft et al. 2024 [27]	Clinical staff + Support staff	Varies - sometimes pharmacist	Multidisciplinary team (Member(s) led)	Unclear	Separation and Shifting
Bailey et al. 2006 [28]	Clinical staff	Unclear	Multidisciplinary team	Unclear	Unclear
Barnes-Boyd et al. 2001 [29]	Clinical staff	Unclear	Multidisciplinary team	Unclear	Separation
Bennett et al. 1988 [30]	Clinical staff	Nurses	Multidisciplinary team (Member(s) led)	Information exchange	Separation
Brown-Johnson et al. 2019 [17]	Clinical staff + Support staff	PCP	Multidisciplinary team (Member(s) led)	Both	Separation and Sharing
Brown et al. 2024 [31]	Clinical staff	Pharmacists	Multidisciplinary team (Member(s) led)	Information transfer	Separation and Shifting
Carriere et al. 2019 [32]	Clinical staff	Physicians	Multidisciplinary team (Member(s) led)	Information exchange	Separation
Chuang et al. 2017 [10]	Clinical staff + Support staff	Unclear	Multidisciplinary team (Member(s) led)	Information transfer	Separation and Shifting
Cliff et al. 2024 [33]	Clinical staff + Support staff	Clinician + Care team coordinator	Multidisciplinary team (Member(s) led)	Information exchange	Separation and Shifting
Corry et al. 2022 [34]	Clinical staff + Support staff	PCP	Multidisciplinary team (Member(s) led)	Information exchange	Separation
Cos et al. 2022 [35]	Clinical staff + Support staff + Community partners	PCP + Behavioural Health Consultant	Multidisciplinary team (Member(s) led)	Both	Separation
Counsell et al. 2009 [36]	Clinical staff + Community partner + Caregiver	PCP	Multidisciplinary team (Member(s) led)	Information exchange	Separation
Day et al. 2011 [37]	Clinical staff + Community partner	Nurses	Multidisciplinary team (Member(s) led)	Unclear	Separation
Deales et al. 2014 [16]	Clinical staff	GP	Multidisciplinary team (Core team, referrals as needed)	Unclear	Separation
Del Toro et al. 1994 [38]	Clinical staff + Community partner	PCP + MSW	Multidisciplinary team (Core team, referrals as needed)	Unclear	Separation
Digiacomo et al. 2010 [39]	Clinical staff	GP	Multidisciplinary team (Core team, referrals as needed)	Information exchange	Separation
Dolovich et al. 2019 [14]	Clinical staff + Community partner + Caregiver	Unclear	Varied type of collaboration	Information transfer	Separation
Dubowitz et al. 2009 [40]	Clinical staff + Community partner	MSW	Multidisciplinary team (Member(s) led)	Information transfer	Separation
Eismann et al. 2023 [41]	Clinical staff + Support staff + Community partners	Paediatrician/PCP + Parenting specialist	Multidisciplinary team (Member(s) led)	Both	Separation
Elley et al. 2008 [42]	Clinical staff	Nurses	Multidisciplinary team (Core team, referrals as needed)	Information transfer	
Feehan et al. 2020 [11]	Clinical staff + Community partner	PCP	Multidisciplinary team (Medical home)	Information exchange	Separation
Ferrer et al. 2009 [43]	Clinical staff + Support staff + Community partners	MA	Multidisciplinary team (Member(s) led)	Information transfer	Shifting
Fiset-Laniel et al. 2020 [44]	Clinical staff	GP + NP	Multidisciplinary team (Member(s) led)	Both	Sharing
Fortuna et al. 2021 [45]	Clinical staff + Support staff + Community partners	Unclear	Multidisciplinary team (Medical home)	Unclear	Unclear
Funderburk et al. 2020 [46]	Clinical staff	Unclear	Multidisciplinary team	Unclear	Unclear
Gannon et al. 2012 [47]	Clinical staff + Support staff	Unclear	Multidisciplinary team	Information exchange	Separation
Graffy et al. 2010 [48]	Clinical staff + Support staff	Unclear	Multidisciplinary team	Unclear	Separation and Shifting
Green et al. 2015 [49]	Clinical staff	GP + Nurse	Multidisciplinary team (Member(s) led)	Information transfer	Separation
Haavet et al. 2021 [50]	Clinical staff	Mental health specialist + PCP	Multidisciplinary team (Member(s) led)	Information exchange	Sharing and Shifting
Hebert et al. 2001 [51]	Clinical staff	Nurses	Multidisciplinary team (Member(s) led)	Information transfer	Separation
Hoffman K et al. 2015 [52]	Clinical staff + Support staff	Unclear	Multidisciplinary team (Member(s) led)	Unclear	Unclear
Hudson et al. 2007 [13]	Clinical staff	Nurses + Health educators	Multidisciplinary team (Member(s) led)	Unclear	Separation
Janse et al. 2016 [53]	Clinical staff + Community partner + Caregiver	GP	Multidisciplinary team (Member(s) led)	Both	Separation
Jaqua et al. 2022 [54]	Clinical staff + Support staff	Resident Physician	Multidisciplinary team (Member(s) led)	Information exchange	Separation
Jennings et al. 2014 [55]	Clinical staff	Nurses	Multidisciplinary team (Member(s) led)	Information exchange	Shifting
Kenny et al. 2023 [56]	Clinical staff + Support staff + Community partners	PCP	Multidisciplinary team (Member(s) led)	Information transfer	Separation and Shifting
Ketola et al. 2001 [57]	Clinical staff	Doctors + Nurses	Multidisciplinary team (Member(s) led)	Both	Separation
Klein et al. 2017 [58]	Clinical staff	PCP + kinesiologist	Multidisciplinary team (Member(s) led)	Unclear	Sharing and Shifting
Lawson et al. 2012 [59]	Clinical staff	FP + NP	Multidisciplinary team (Member(s) led)	Information exchange	Sharing and Shifting
Lee et al. 2023 [60]	Clinical staff + Support staff	PCP	Multidisciplinary team (Member(s) led)	Information transfer	Separation
Levy et al. 2017 [61]	Clinical staff + Community partners	Mental health/developmental specialist	Multidisciplinary team (Member(s) led)	Information exchange	Separation
Lewin et al. 2019 [62]	Clinical staff + Community partner	Unclear	Multidisciplinary team	Information exchange	Separation
Looman et al. 2016 [63]	Clinical staff + Community partner + Caregiver	GP + NP	Multidisciplinary team (Member(s) led)	Both	Separation
Marfo et al. 2016 [64]	Clinical staff + Support staff	Medicine Counter Assistant	Multidisciplinary team (Member(s) led)	Information transfer	Shifting
Matthew et al. 2023 [65]	Clinical staff + Support staff + Community partners	Unclear	Multidisciplinary team (Core team, referrals as needed)	Unclear	Sharing
McNamara et al. 2020 [66]	Clinical staff	GP + Pharmacists	Multidisciplinary team (Member(s) led)	Information transfer	Shifting
Metzelthin et al. 2013 [8]	Clinical staff + Caregiver	Practice nurse	Multidisciplinary team (Member(s) led)	Information exchange	Separation
Metzelthin et al. 2013 [67]	Clinical staff + Caregiver	Practice nurse	Multidisciplinary team (Member(s) led)	Information exchange	Separation
Miller et al. 2021 [68]	Clinical staff + Support staff	Advanced practice providers (physician assistants, clinical pharmacists)	Multidisciplinary team (Member(s) led)	Information transfer	Separation
Misra-Hebert et al. 2018 [69]	Clinical staff + Support staff	Physicians	Multidisciplinary team (Member(s) led)	Information transfer	Separation and Shifting
Muench et al. 2015 [1]	Clinical staff + Support staff	Physicians	Multidisciplinary team (Member(s) led)	Information transfer	Separation and Shifting
Negriff et al. 2022 [70]	Clinical staff	Paediatricians	Multidisciplinary team (Member(s) led)	Both	Separation
Nguyen et al. 2020 [15]	Clinical staff + Support staff	Unclear	Multidisciplinary team	Information exchange	Separation
Parker et al. 2022 [71]	Clinical staff + Support staff	Practice nurse	Multidisciplinary team (Member(s) led)	Information transfer	Sharing and Shifting
Passmore et al. 2024 [72]	Clinical staff + Support staff	RN + Clinician + Admin	Multidisciplinary team (Member(s) led)	Both	Separation
Peixoto et al. 2024 [73]	Clinical staff + Community partner	Family Physician	Multidisciplinary team (Member(s) led)	Both	Separation
Perell et al. 2006 [74]	Clinical staff + Support staff	Unclear	Multidisciplinary team	Information exchange	Separation
Perrin et al. 2018 [75]	Clinical staff + Community partner + Caregiver	Unclear	Multidisciplinary team	Information exchange	Sharing and Shifting
Ramos et al. 2021 [76]	Clinical staff	Community pharmacist + GP	Multidisciplinary team (Member(s) led)	Both	Shifting
Rennie et al. 2015 [77]	Clinical staff	Nurses	Multidisciplinary team (Member(s) led)	Both	Sharing and Shifting
Rosana et al. 2021 [78]	Clinical staff + Community partner	Nurses	Multidisciplinary team (Member(s) led)	Information transfer	Separation
Rowe et al. 2012 [79]	Clinical staff	Nurses + Family medicine resident	Multidisciplinary team (Member(s) led)	Information transfer	Sharing and Separation
Ruikes et al. 2016 [80]	Clinical staff + Community partner	GP	Multidisciplinary team (Member(s) led)	Information exchange	Separation
Schlauderaff et al. 2017 [81]	Clinical staff + Support staff	Medical assistant	Multidisciplinary team (Member(s) led)	Information transfer	Shifting and Separation
Schor et al. 2019 [82]	Clinical staff + Community partners	Nurses + Physicians	Multidisciplinary team (Member(s) led)	Information exchange	Separation
Selfridge et al. 2025 [83]	Clinical staff + Support staff	Nurse Coordinator + Nurse	Multidisciplinary team (Member(s) led)	Both	Separation
Siwik et al. 2012 [84]	Clinical staff	Physicians	Multidisciplinary team (Member(s) led)	Both	Separation and Sharing
Smith et al. 2019 [85]	Clinical staff + Support staff	MA	Multidisciplinary team (Member(s) led)	Information transfer	Shifting and Separation
SmitsGH et al. 2022 [86]	Clinical staff	Practice nurse + GP	Multidisciplinary team (Member(s) led)	Information transfer, Information exchange	Shifting and Separation
Smits GH et al. 2023 [87]	Clinical staff	Discussed below	Multidisciplinary team (Member(s) led)	Information transfer, Information exchange	Unclear
Sookaneknun et al. 2010 [88]	Clinical staff	Pharmacists/PharmD students + Nurses	Multidisciplinary team (Member(s) led)	Information transfer	Shifting and Separation
Stuck et al. 2015 [89]	Clinical staff	Nurse counsellors	Multidisciplinary team (Member(s) led)	Information exchange	Separation
Sturmberg et al. 1999 [90]	Clinical staff	GP	Multidisciplinary team (Member(s) led)	Information transfer	Separation
Tapsell et al. 2017 [91]	Clinical staff	Unclear	Multidisciplinary team	Information transfer	Separation
Thomas et al. 2014 [92]	Clinical staff	Pharmacists	Multidisciplinary team (Member(s) led)	Both	Shifting
Thomas et al. 2015 [93]	Clinical staff	Health Service Providers	Multidisciplinary team (Member(s) led)	Information exchange	Separation
Vagholkar et al. 2024 [94]	Clinical staff	Mandarin speaking practice nurse + GP	Multidisciplinary team (Member(s) led)	Both	Separation
Valaitis et al. 2020 [95]	Clinical staff + Support staff + Community partners	PCP	Multidisciplinary team (Member(s) led)	Both	Sharing
Vedel et al. 2009 [96]	Clinical staff + Community partner	GP	Multidisciplinary team (Member(s) led)	Both	Sharing and Separation
Vermunt et al. 2012 [97]	Clinical staff	Unclear	Multidisciplinary team	Information exchange	Sharing and Shifting
Warmels et al. 2017 [98]	Clinical staff	Nurses	Multidisciplinary team (Member(s) led)	Information exchange	Shifting
Williams et al. 2007 [99]	Clinical staff	GP	Multidisciplinary team (Member(s) led)	Both	Separation
Wilmot et al. 1984 [100]	Clinical staff + Community partner	Unclear	Multidisciplinary team	Both	Sharing and Separation
Wiltshire et al. 2023 [101]	Clinical staff + Support staff	Nurse educator + Physician	Multidisciplinary team (Member(s) led)	Information transfer	Separation
Wittink et al. 2019 [102]	Clinical staff	PCP	Multidisciplinary team (Member(s) led)	Both	Separation
Woodberry et al. 2025 [103]	Clinical staff	Behavioural Health Clinician + PCP	Multidisciplinary team (Member(s) led)	Both	Separation and Shifting
Wooten et al. 2017 [104]	Clinical staff + Community partner	Physicians	Multidisciplinary team (Member(s) led)	Information exchange	Sharing and Shifting
Wright et al. 2021 [105]	Clinical staff + Support staff	Health Service Providers	Multidisciplinary team (Member(s) led)	Information transfer	Separation

### Study characteristics

The included studies were conducted in various regions: North America (*n* = 56), UK/Europe (*n* = 24), Australia/New Zealand (*n* = 9), Asia (*n* = 1), Africa (*n* = 1), Middle East (*n* = 1), South America (*n* = 1) and unclear (*n* = 1). Most studies were performed in primary care clinics (*n* = 71), followed by those conducted in university/community settings (*n* = 12), pharmacies (*n* = 3), academic clinics (*n* = 3), medical home (*n* = 1); combined (clinic/pharmacies/community - *n* = 1; clinic/community – *n* = 3). The study designs varied: cross-sectional studies (*n* = 8), qualitative and descriptive studies (*n* = 12), randomized controlled trials (*n* = 14), mixed-method studies (*n* = 12), non-randomised controlled trials (*n* = 6), quasi-experimental studies (*n* = 9), reports (*n* = 4), observational (*n* = 2), retrospective analyses (*n* = 10), feasibility studies (*n* = 5), case study (*n* = 4), cost analysis (*n* = 1), and narrative analyses (*n* = 1).

The research methods used in the studies included self-reports (i.e. questionnaires, surveys and interviews) (*n* = 17) and observations (*n* = 9) as well as record data and physiological measurements (*n* = 24), combined (physiological measurements and self reports- *n* = 15; physiological measurements and records review – *n* = 8; observational and self reports -*n* = 7; physiological measurements, observational and self reports – *n* = 7; physiological measurements, records review and self reports – *n* = 3; records review and self reports -*n* = 3), records review (*n* = 13); unclear (*n* = 1).

### Types of preventive health services

The studies covered various types of preventive health services: maternal, adolescent and child health (*n* = 1); screening, health promotion/weight management (*n* = 36); geriatric care (*n* = 16); mental health (*n* = 10); paediatric care (*n* = 10); prevention and management of high-risk lifestyle activities (*n* = 12); and general preventive services (*n* = 12). The distribution of included studies by type of preventive health service was recorded in Table 2. Definitions for each type of services were included above.

**Table 2:** Distribution of included studies by type of preventive health services

Type of Preventive Health Service	Studies
Screening and Health Promotion/Weight Management (*n* = 36)	Aboueid et al. 2018 [22], Aboueid et al. 2018 [23], Bennett et al. 1988 [30], Brown et al. 2024 [31], Carriere et al. 2019 [32], Chuang et al. 2017 [10], Day et al. 2011* [37], Deales et al. 2014 [16], Digiacomo et al. 2010 [39], Fiset-Laniel et al. 2020 [44], Gannon et al. 2012 [47], Graffy et al. 2010 [48], Hudson et al. 2007 [13], Jaqua et al. 2022 [54], Klein et al. 2017 [58], Lawson et al. 2012 [59], Marfo et al. 2016 [64], McNamara et al. 2020 [66], Miller et al. 2021 [68], Nguyen et al. 2020 [15], Parker et al. 2022 [71], Ramos et al. 2021 [76], Rennie et al. 2015 [77], Rosana et al. 2021 [78], Rowe et al. 2012 [79], Schlauderaff et al. 2017 [81], Selfridge et al. 2025 [83], Siwik et al. 2012 [84], Smith et al. 2019 [85], Sookaneknun et al. 2010 [88], Sturmberg et al. 1999 [90], Tapsell et al. 2017 [91], Thomas et al. 2014 [92], Thomas et al. 2015 [93], Vermunt et al. 2012 [97], Wright et al. 2021 [105]
Geriatric Care (*n* = 16)	Abbasi et al. 2021 [9], Abrams et al. 2015* [24], Corry et al. 2022 [34], Counsell et al. 2009 [36], Dolovich et al. 2019 [14], Elley et al. 2008 [42], Hebert et al. 2001 [51], Looman et al. 2016 [63], Metzelthin et al. 2013 [8], Metzelthin et al. 2013 [67], Perell et al. 2006 [74], Ruikes et al. 2016 [80], Stuck et al. 2015 [89], Valaitis et al. 2020 [95], Vedel et al. 2009 [96], Williams et al. 2007 [99]
General Preventive Services (*n* = 12)	Ashcroft et al. 2024 [26], Bailey et al. 2006 [28], Brown-Johnson et al. 2019 [17], Cliff et al. 2024 [33], Fortuna et al. 2021 [45], Hoffman K et al. 2015 [52], Matthew et al. 2023 [65], Misra-Hebert et al. 2018 [69], Passmore et al. 2024 [72], Perrin et al. 2018 [75], Schor et al. 2019 [82], Wiltshire et al. 2023 [101]
Prevention/Management of High-Risk Lifestyle Activities (*n* = 12)	Del Toro et al. 1994 [38], Ferrer et al. 2009 [43], Green et al. 2015 [49], Jennings et al. 2014 [55], Ketola et al. 2001 [57], Lee et al. 2023 [60], Muench et al. 2015 [1], Peixoto et al. 2024 [73], SmitsGH et al. 2022 [86], Smits GH et al. 2023 [87], Vagholkar et al. 2024 [94], Wooten et al. 2017 [104]
Pediatric Care (*n* = 10)	Ammerman et al. 2022 [25], Arthur NSM et al. 2023 [26], Barnes-Boyd et al. 2001 [29], Dubowitz et al. 2009 [40], Eismann et al. 2023 [41], Feehan et al. 2020 [11], Kenny et al. 2023* [56], Negriff et al. 2022 [70], Warmels et al. 2017 [98], Wilmot et al. 1984 [100]
Mental Health (*n* = 10)	Abrams et al. 2015* [24], Cos et al. 2022 [35], Day et al. 2011* [37], Funderburk et al. 2020 [46], Haavet et al. 2021 [50], Janse et al. 2016 [53], Kenny et al. 2023* [56], Levy et al. 2017 [61], Wittink et al. 2019 [102], Woodberry et al. 2025 [103]
Maternal, Adolescent and Child Health (*n* = 1)	Lewin et al. 2019 [62]

* Indicates studies addressing more than one type of preventive service (Abrams et al. 2015 [24], Day et al. 2011 [37], Kenny et al. 2023 [56]), resulting in a total category count (*n* = 97) exceeding the number of included studies (*n* = 94)

### Team composition and tasking

Regarding team composition, the membership varied as follows: clinical staff only (*n* = 38) [13, 16, 28–32, 39, 42, 44, 46, 49–51, 55, 57–59, 66, 70, 76, 77, 79, 84, 86, 88–92, 94, 97–99, 102, 103]; both clinical and support staff (*n* = 24) [1, 10, 15, 17, 26, 27, 33, 34, 47, 52, 54, 60, 64, 68, 69, 71, 72, 74, 81, 83, 85, 101, 105], clinical staff and community partners (*n* = 16) [11, 22–24, 37, 38, 40, 61, 62, 73, 78, 82, 96, 100, 104, 106]; clinical staff, community partners and support staff (*n* = 7) [41, 43, 45, 56, 65, 95]; clinical staff, community parners and caregivers (*n* = 7) [9, 14, 25, 36, 53, 63, 75]; clinical staff and caregivers (*n* = 2) [8, 67]. In some TBC models [9, 11, 14, 22–25, 35–38, 40, 41, 43, 45, 53, 56, 61–63, 65, 73, 75, 78, 80, 82, 95, 96, 100, 104], community partners assumed diverse roles and responsibilities depending on their expertise and experiences. These include participation from medical social workers and case managers as well as more informal roles such as community volunteers and helpline resources. Notably, therapists and teachers were important contributing team members in paediatric mental health interventions [61]. Similarly, patients and caregivers played integral roles. Under Abbasi et al.’s integrated Seniors’ Community Hub model, care planning involved active discussions between patients, caregivers and health care providers to prioritise care tasks and develop customised intervention plans [9]. In Ammerman et al.’s integrated behavioural health model, deliberate efforts were made to engage and build relationships with caregivers to educate, motivate and encourage them to participate in future well-child visits [25].

In terms of team tasking, different methods were employed (when mentioned), including task separation (*n* = 50) [8, 9, 11, 13–16, 22, 23, 25, 29, 30, 32, 34–41, 47, 49, 51, 53, 54, 57, 60–63, 67, 68, 70, 72–74, 78, 80, 82, 83, 89–91, 93, 94, 99, 101, 102, 105], task sharing (*n* = 3) [44, 65, 95] and task shifting (*n* = 7) [43, 55, 64, 66, 76, 92, 98]. Combined separation and sharing (*n* = 5) [17, 79, 84, 96, 100], separation and shifting (*n* = 13) [1, 10, 31, 33, 48, 56, 69, 81, 85, 86, 88, 103], sharing and shifting (*n* = 9) [24, 50, 58, 59, 71, 75, 77, 97, 104]. For studies employing task separation [1, 8–11, 13–17, 22, 23, 25, 27, 29–41, 47–49, 51, 53, 54, 56, 57, 60–63, 67–70, 72–74, 78–86, 88–91, 93, 94, 96, 99–103, 105] we observed that the roles of each member were well-defined with minimal overlap among team members. In these studies, intervention tasks were allocated based on the standard roles and skillsets of each member. For example, in Miller et al., [68] programme support assistants continued to handle routine administrative tasks, such as providing enrolment materials to veterans and managing patient inquiries. However, no new roles were assumed; their usual task was integrated into a standardised workflow alongside the main preventive care activities led by other clinical staff [68].

Task separation is particularly useful for standardized preventive care tasks such as vaccinations and screenings. Strict separation can also limit flexibility, making it difficult to adapt in cases of workforce shortages and potentially contributing to fragmented care [107]. By comparison, task sharing [44, 65, 95] allows multiple team members to jointly perform or alternate responsibilities, enhancing care continuity and reducing individual provider workload. This model fosters interprofessional collaboration and learning but requires clear protocols to prevent redundancy or miscommunication. Additionally, highly specialized tasks may not be well-suited for sharing if they require advanced expertise [108]. In other studies [24, 43, 50, 55, 58, 59, 64, 66, 71, 75–77, 92, 97, 98, 104] task shifting, which involves transferring responsibilities from higher-trained professionals to those with less training under appropriate supervision, can improve workforce efficiency and expand access to care.

For instance, in *Bennett et al.,* community pharmacists undertook the novel role of screening for early signs and symptoms of mild cognitive impairment during routine medication dispensing, with results communicated to general practitioners in primary health care centres [30] This approach is particularly valuable in resource-limited settings; however, it necessitates structured training and support to maintain quality and prevent excessive workload stress on lower-level providers [109, 110] Sometimes, task shifting can simultaneously occur with task sharing, where one team member would take on the role of another team member, alternatingly providing care for patients [24, 50, 58, 59, 71, 75, 77, 97, 104]. Higher-expertise members would provide consultative services and support to those conducting these intervention tasks [25, 61].

### TBC delivery and leadership

**Fig. 2:**
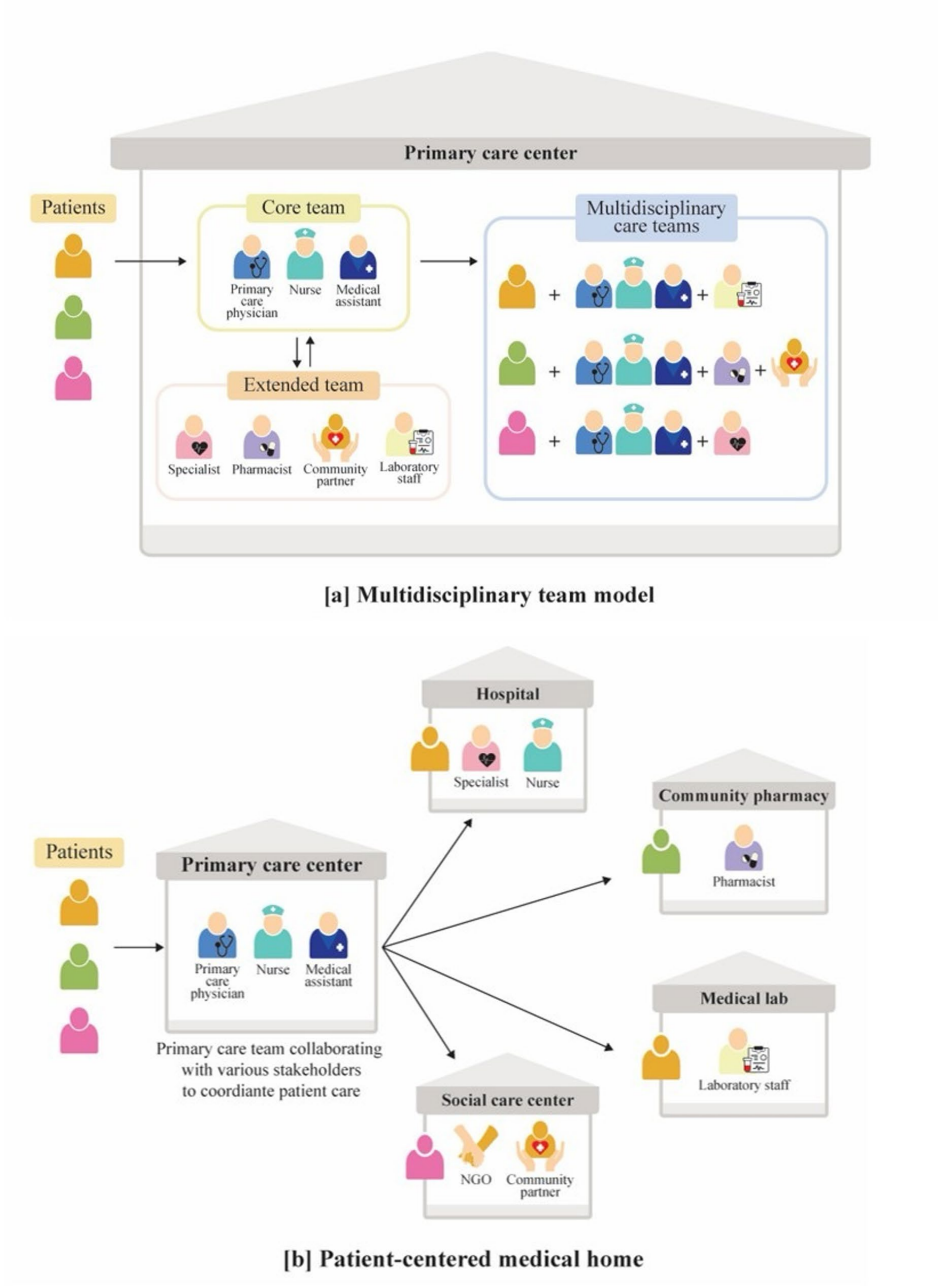
Pictogram showing the different team-based care models used. **A**: General Multidisciplinary team approach; **B**: Patient-Centred Medical Home

TBC were delivered as either multidisciplinary team (MDT) (*n* = 90) including member-led (*n* = 69) [1, 8–10, 13, 17, 27, 30–37, 40, 41, 43, 44, 49–61, 63, 64, 66–73, 76–90, 92–96, 98, 99, 101–105], general MDT (*n* = 16) [15, 22–25, 28, 29, 46–48, 62, 74, 75, 91, 97, 100], and core team with referrals as needed (*n* = 5) [16, 38, 39, 42, 65]; or medical homes (*n* = 3) [11, 26, 45]; mixed models (*n* = 1) [14] (Fig. 2). The most commonly employed approach was the use of an MDT, which comprised more than two members who worked collaboratively within a team setting. The MDT models varied in design and function, with some studies encouraging collaborative decision-making and discussion among team members to develop customised care plans for patients [15, 50, 78, 80] while others [1, 34, 37, 58] utilised a sequential workflow where each team member carried out their assigned roles independently by ‘handing over’ tasks. Within MDT, a collaboration strategy commonly employed involved member(s)-led interventions. Another prevalent model involved a core team with referrals to other health care providers as needed [16, 38, 39, 42, 65].

Primary care provider-led models—including those led by general practitioners/primary care providers, nurse practitioners/practice nurse, and resident physicians—were the most common (*n*= 33) [1, 8, 9, 11, 16, 17, 32, 34, 36, 39, 53, 54, 56, 59, 60, 67, 69, 71, 73, 80, 84, 86, 90, 93–96, 99, 102, 104, 105]. Nurse-led models were also prevalent (*n*= 10) [30, 37, 42, 51, 55, 77, 78, 83, 89, 98]. Less commonly, leadership was attributed to medical assistants (*n* = 4) [43, 64, 81, 85], pharmacists (*n* = 2) [31, 92], mental health specialist (*n* = 1) [61] and medical social workers (*n* = 1) [40].

Paired-member led models include collaboration between: doctor and nurses (*n* = 3) [49, 57, 101], PCP and mental health specialists (*n* = 3) [35, 50, 103], PCP and MSW (*n* = 1) [38], GP and pharmacists (*n* = 2) [66, 76], pharmacists and nurses (*n* = 1) [88], PCP and parenting specialist (*n* = 1) [41], PCP and kinesiologist (*n* = 1) [58], nurses and educator/resident (*n* = 2) [13, 79], admin/support coordinator and clinician (*n* = 2) [33, 72].

Finally, the Patient-Centred Medical Home (PCMH) model, defined as a primary care organisation model that delivers core functions of primary health care, offered a clear structure and integration approach to TBC. In PCMH model, the primary care physician acts as the leader and main coordinator of care between patients and the rest of the care team.

Each patient is assigned to a primary care physician and a primary care team, which at minimum, includes the physician, case manager and administrative personnel and will be expanded to include other specialists, nursing, allied health workers at the practice level as required. These practices also had access to additional resources such as data coordinators and care managers to support preventive care services for their patients.

By comparison, member-led interventions do not require the leader/main task coordinator to be a physician; it could be nurses, pharmacists, medical assistants, or other members of the healthcare team. The degree of coordination is often not as extensive as that in PCMH. In PCMH, the primary care physician is the main point of coordination, and patients gain access to extensive additional resources as required, including hospital services, community pharmacy, social care centers, and medical labs.

The various collaboration strategies utilized in the studies were similar to the levels of collaboration and integration proposed by Doherty et al [111]. The most basic model involves team members working in separate facilities, and having minimal communication where the collabration includes need-based referrals. A mid-level form of collaboration is colocation where team members operate in close proximity with differing degrees of onsite interaction. The highest level of team-based care is full integration, characterized by unified facilities, consolidated systems for scheduling and billing, and a blending of distinct professional cultures [112]. MDTs are structured to address specific, often complex medical conditions through diverse expertise, while PCMHs aim to provide comprehensive, patient-focused care across a broad spectrum of health needs. Both models have unique strengths and challenges, and their adoption depends on the healthcare setting and the population being served.

### Team communication

The TBC models employed different communication strategies: information exchange (*n* = 27) [8, 11, 15, 24, 30, 32–34, 36, 39, 47, 50, 54, 55, 59, 61, 62, 67, 74, 75, 80, 82, 89, 93, 97, 98, 104]; information transfer (*n* = 25) [1, 10, 14, 31, 40, 42, 43, 49, 51, 56, 60, 64, 66, 68, 69, 71, 78, 79, 81, 85, 88, 90, 91, 101, 105]; both information exchange and transfer (*n* = 24) [17, 35, 41, 44, 53, 57, 63, 70, 72, 73, 76, 77, 83, 84, 86, 87, 92, 94–96, 99, 100, 102, 103]. In information transfer, team members gathered information and communicated to others through various channels such as referral notes, electronic records and professional reports. Information transfer is commonly used for straightforward, standardized preventive tasks such as screenings and vaccinations [81, 105]. This method facilitates efficiency by reducing the need for direct provider interaction while ensuring that essential clinical information is documented and relayed through electronic health records (EHRs), referral notes, or standardized reports [64, 81, 105].

On the contrary, information exchange involved multiple members engaging in shared decision-making activities. It is typically employed for more complex interventions, such as geriatric care needs assessments, chronic disease management, and integrated behavioral health interventions [24]. Complex scenarios often involve multiple interrelated factors—such as polypharmacy in older adults or the interplay of psychological and physical health—which necessitate real-time discussion among clinicians, social workers, caregivers, and patients [63]. To facilitate these discussions, face-to-face meetings, case conferences, or real-time consultations are essential for integrating diverse expertise and ensuring that care plans remain holistic and patient-centered. In many cases, both types of communication were utilized.

Lastly, colocation (*n* = 5) [24, 37, 50, 61, 104] emerged as one of the strategies to enhance communication where health care specialists shared physical space with PCPs. Subsequently, they provided direct resources for referral and consultation. This model facilitated joint consultations and diagnostic assessments, as demonstrated in a couple of studies.

### TBC outcomes

TBC outcomes included: TBC delivery (documentation and communication) (*n* = 1); TBC implementation outcomes (fidelity, adoption) (*n* = 2); TBC effectiveness (job satisfaction and preventive service uptake (*n* = 5); perceptions and experiences of health care providers with TBC (roles, team work and performance) (*n* = 5); perceptions of health care providers on teamwork and team performance (*n* = 3); and patients’ perceptions and experiences of TBC (*n* = 6) (Table 3).

**Table 3:** TBC related outcomes described in studies

TBC outcomes	Description
**TBC delivery**	
Communication [46]	• Overall communication process
	• Accessibility to primary care team members
	• Ability to listen and openness to others’ perspectives
	• Value of in-person feedback
Documentation [46]	• Ability to identify relevant information in these notes
	• Value of EMR including notifications and suicide risk alerts
**TBC implementation**	
Fidelity to TBC [8]	• Inter-disciplinary care team adherence to the intervention
	• Adherence to the team protocol vs. clinical protocol
Adoption [69]	• Level of uptake of team-based practice models
Effectiveness [13] - Health - Job satisfaction and burden [12, 27, 38, 59]	• Screening uptake rates for colorectal cancer through behavioural counselling, health risk assessment protocols or questionnaires and reminder systems
	• Providers’ satisfaction with TBC
	• Perception and impact of TBC on job burden, job satisfaction, burnout and work-life balance
**Care providers’ perceptions and experiences of TBC**	
Perceived role [10, 17, 28, 48, 69]	• Perception of roles and scope of practice of team members
	• Perceived control over their professional practice
	• Role distinction and value
Perceptions and experiences with TBC [10, 28, 48, 61, 98]	• Overall experience with TBC
	• Perception of team structure and functioning
	• Experiences with TBC implementation
	• Perceptions of colocation models
	• Perceptions of flexible workflow
	• Perception of effectiveness of TBC
Teamwork and team performance [45, 46, 95]	• Perceptions of patient care, professional experience and teamwork
	• Perception of work competency of team members
	• Risk concerns when other team members are involved
	• Experiences regarding teamwork
	• Team performance
**Patients’ TBC perception and experience of TBC**	
Views about the TBC [69, 98]	• Perceptions of TBC model
	• General experience with the TBC model and perceptions of its effectiveness
Satisfaction with TBC [8, 17, 97, 98]	• Satisfaction with TBC

*EMR* Electronic medical record, *NP* Nurse practitioner, *TBC* Team-based care

## Discussion

### Summary

This scoping review of 92 studies found that TBC in preventive health is highly adaptable, involving diverse team members including clinical staff, support roles, and community partners. Most models used multidisciplinary teams or patient-centered medical homes. Common task delegation strategies included task separation and shifting while communication strategies included information exchange and transfer.

### Comparison with existing literature

#### Team composition and role flexibility

Consistent with *Bates et al.* and *Frikha et al.* [113, 114], who reported the growing importance of interdisciplinary teams and expanded non-physician roles, our review identifies even greater task flexibility. Medical assistants and pharmacists were shown to take on screening and coordination roles, while patients and caregivers were integrated as active team members. This aligns with the Starfield Summit report [115] which emphasized the adaptability of primary care teams to local workforce availability and patient needs. In particular, the inclusion of behavioral health professionals, case managers, and clerical staff demonstrates how preventive TBC models expand staffing beyond traditional clinical roles. This echoes Sørensen et al [116], who highlighted the need for clearly defined yet dynamic team roles based on patient complexity and team context.

#### Tasking approaches

Previous literature has recognized the value of task shifting to optimize workforce distribution, especially in low-resource settings. Our review adds granularity by characterizing models of task separation, task sharing, and hybrid tasking which is in line with findings from *Li et al.* [117] who emphasized the importance of aligning tasks with competencies across roles, particularly to reduce duplication and ensure patient-centeredness. Our results further suggest that task reallocation depends on clarity in responsibility, a theme also emphasized in the Starfield Summit report [115], where ambiguity in role definition was linked to team inefficiency and burnout.

#### Communication and integration

Besides the importance of EHRs in streamlining communication [113, 114], our review also highlights dual modes of communication: asynchronous data transfer via EMRs and synchronous exchange through face-to-face meetings or joint consultations. Similarly, the Starfield Summit report and *Li et al.* also noted that real-time collaboration—whether colocated or virtual—is key to managing the temporal and spatial separation common in primary care teams [115, 117]. In particula, integrated information systems must be tailored to support fluid communication and coordination, especially when roles span across organizational boundaries [117].

#### Care models and leadership

Besides focusing on physician-led models [113, 114], our review reveals a broader spectrum of leadership structures, including nurse-led, allied health-led, and shared leadership models in preventive services. This supports *Wohler & Liaw et al.*.’s argument that leadership in TBC should be adaptable, with distributed authority based on task complexity and contextual demands [115].

#### Outcomes and implementation

In addition to chronic disease-oriented metrics (e.g., HbA1c, blood pressure) discussed in other reviews [113, 114], our review also reports implementation and experiential outcomes such as team satisfaction, role clarity, and stakeholder perceptions. Exploring these dimensions further can deepen interprofessional understanding, strengthen trust, and illuminate key barriers and facilitators to effective and sustainable implementation of team-based care [115, 117].

### Strength and limitations

This scoping review is the first of its kind to examine a wide range of existing literature on the implementation of TBC in preventive care settings. To ensure adequate surveying of the existing literature, the help of a librarian was enlisted to aid in search term development and optimization. In addition, broad inclusion criteria, encompassing various study designs such as cross-sectional, qualitative and descriptive, randomized controlled trials, mixed-method, were deliberately chosen to ensure the review captured as many relevant studies in primary care as possible. In addition, independent screening and data extraction by two authors minimized risks of human errors.

A notable limitation of this study is the number of irretrievable texts, accounting for approximately 50 out of the 379 sources. This was due to lack of access to the articles. This may lead to an insufficient understanding and analysis of TBC models. However, a considerable proportion of these studies were dated over 10 years, which may limit their direct relevance to the current literature landscape.

### Future directions/research implications

Future studies on team-based care should include detailed reporting of team composition, collaboration types, communication strategies, and intra-team quality assurance methods. Such transparency is essential for understanding the mechanisms through which TBC delivers its outcomes. Additionally, more rigorous study designs—particularly randomized controlled trials—are needed to strengthen the evidence base. Finally, future research should focus on developing a systematic classification of TBC models, supported by comprehensive and validated evaluation tools.

## Conclusions

Health systems are increasingly prioritizing preventive care over reactive treatment models to improve population health and reduce long-term costs [118]. Evidence shows that proactive interventions enhance equity, access, and system efficiency, particularly in underserved communities [118]. These trends underscore the importance of reviewing how TBC is deployed in preventive services. Our review contributes to this by mapping how TBC is structured, coordinated, and implemented in preventive health contexts—highlighting its role in advancing modern primary care priorities.

We also have highlighted the diverse applications of TBC, demonstrating its adaptability across various clinical settings, populations and preventive care conditions. In conclusion, while TBC holds promise for enhancing preventive care services, addressing the identified limitations and advancing research are crucial for optimising its implementation in preventive care.

## Supplementary Information

Supplementary Material 1.

Supplementary Material 2

Supplementary Material 3.

## Data Availability

Not applicable.

## Abbreviations

TBC: Team-based care
MDT: Multidisciplinary team
PRISMA ScR: Preferred Reporting Items for Systematic Reviews and Meta-Analyses–Extension for Scoping Reviews
EMR: Electronic medical record
FP: Family physician
GP: General practitioner
PCP: Primary care provider
NP: Nurse practitioner
MA: Medical assistant
RN: Registered nurse
