# Supplementary material for: Exploring diverse applications of team-based care in preventive medicine: a scoping review
Source: BMC Prim Care. 2026 Apr 29;27:232. doi: 10.1186/s12875-026-03311-8 (PMC13267235; doi:10.1186/s12875-026-03311-8)
Supplement: Supplementary file 3 — Supplementary Material 3. [file 12875_2026_3311_MOESM3_ESM.docx]

**Appendix S1.** Full search strategy.

| **PCC elements** | **Key words** | **Search terms** |
| --- | --- | --- |
| Population | Preventive health services | (**“Preventive health services”** OR  **“Preventive medicine”** OR  “Mass screening” OR  Screen*OR Prevent* OR  “Early detection” OR  “Immunization”)  **AND** |
| Concept | Integrated care | (**“Delivery of health care, integrated”** OR  **“Practice patterns, nurses”** OR  **“Practice patterns, pharmacists”** OR  **“Interprofessional relations”** OR  **“Patient care team”** OR  “Team-based” OR  “Integrated care” OR  “Collaborative care” OR  “Pharmacist-led” OR  “Nurse-led” OR  “Interprofessional team” OR  “Teamwork” OR  “Pharmacist-physician” OR  “Nurse-physician” OR  “Interdisciplinary care” OR  “Multidisciplinary care” OR  “Primary-care team”)  **AND** |
| Comparison | N/A | N/A |
| Context | Primary care | (**“Primary care”** OR  **“Primary health care”** OR  “General practice” OR  “GP” OR  “Family practice” OR  “Family medicine” OR  “Family doctor” OR  “Family physician”) |

**PubMed Executable Search String**

((((((((((preventive health services[MeSH Major Topic]) OR (preventive medicine[MeSH Major Topic])) OR (mass screening[MeSH Major Topic])) OR (screen*[Title/Abstract])) OR (prevent*[Title/Abstract])) OR ("early detection"[Title/Abstract])) OR ("immunization"[Title/Abstract])) OR ("preventive medicine"[Title/Abstract])) OR ("preventive health service"[Title/Abstract])) AND (((((((((((((((((delivery of health care, integrated[MeSH Major Topic]) OR (Patient Care Team[MeSH Major Topic])) OR (practice patterns, nurses[MeSH Major Topic])) OR (practice patterns, pharmacists[MeSH Major Topic])) OR (interprofessional relations[MeSH Major Topic])) OR ("Patient care team"[Title/Abstract])) OR ("team-based"[Title/Abstract])) OR ("integrated care"[Title/Abstract])) OR ("Collaborative care"[Title/Abstract])) OR ("pharmacist-led"[Title/Abstract])) OR ("teamwork”[Title/Abstract])) OR ("primary-care team”[Title/Abstract])) OR ("nurse-physician"[Title/Abstract])) OR ("pharmacist-physician”[Title/Abstract])) OR ("Nurse-led"[Title/Abstract])) OR ("interprofessional team"[Title/Abstract])) OR ("interdisciplinary care"[Title/Abstract]))) AND ((((((((((primary care[MeSH Major Topic]) OR (primary health care[MeSH Major Topic])) OR ("primary health care"[Title/Abstract])) OR ("general practice"[Title/Abstract])) OR ("GP"[Title/Abstract])) OR ("Family practice"[Title/Abstract])) OR ("family medicine"[Title/Abstract])) OR ("primary care"[Title/Abstract])) OR ("family doctor"[Title/Abstract])) OR ("family physician"[Title/Abstract]))
